# Genetic disruption of *Ano5* in mice does not recapitulate human *ANO5*-deficient muscular dystrophy

**DOI:** 10.1186/s13395-015-0069-z

**Published:** 2015-12-21

**Authors:** Jing Xu, Mona El Refaey, Li Xu, Lixia Zhao, Yandi Gao, Kyle Floyd, Tallib Karaze, Paul M. L. Janssen, Renzhi Han

**Affiliations:** Department of Surgery, Davis Heart and Lung Research Institute, Biomedical Sciences Graduate Program, Biophysics Graduate Program, The Ohio State University Wexner Medical Center, Columbus, OH 43210 USA; Department of Physiology and Cell Biology, Department of Internal Medicine, Davis Heart and Lung Research Institute, The Ohio State University Wexner Medical Center, Columbus, OH 43210 USA

**Keywords:** Anoctamin 5, Heart, Muscular dystrophy, Skeletal muscle, TMEM16E

## Abstract

**Background:**

Anoctamin 5 (*ANO5*) is a member of a conserved gene family (TMEM16), which codes for proteins predicted to have eight transmembrane domains and putative Ca^2+^-activated chloride channel (CaCC) activity. It was recently reported that mutations in this gene result in the development of limb girdle muscular dystrophy type 2L (LGMD2L), Miyoshi myopathy type 3 (MMD3), or gnathodiaphyseal dysplasia 1 (GDD1). Currently, there is a lack of animal models for the study of the physiological function of *Ano5* and the disease pathology in its absence.

**Results:**

Here, we report the generation and characterization of the first *Ano5*-knockout (KO) mice. Our data demonstrate that the KO mice did not present overt skeletal or cardiac muscle pathology at rest conditions from birth up to 18 months of age. There were no significant differences in force production or force deficit following repeated eccentric contractions between wild type (WT) and KO mice. Although cardiac hypertrophy developed similarly in both KO and WT mice after daily isoproterenol (ISO, 100 mg/kg) treatment via intraperitoneal injection for 2 weeks, they were functionally indiscernible. However, microarray analysis identified the genes involved in lipid metabolism, and complement pathways were altered in the KO skeletal muscle.

**Conclusions:**

Taken together, these data provide the evidence to show that genetic ablation of *Ano5* in C57BL/6J mice does not cause overt pathology in skeletal and cardiac muscles, but *Ano5* deficiency may lead to altered lipid metabolism and inflammation signaling.

**Electronic supplementary material:**

The online version of this article (doi:10.1186/s13395-015-0069-z) contains supplementary material, which is available to authorized users.

## Background

The TMEM16 family of membrane proteins, also known as anoctamins, plays crucial roles in a variety of physiological processes including ion transport, phospholipid scrambling, as well as regulating other ion channels. Members of this family share common structural characteristics including eight transmembrane domains, a re-entrant loop between the fifth and sixth transmembrane domains forming the channel pore [1], and a unique sequence motif called the annotated domain of unknown function 590 (DUF590) [2, 3]. Among this family, *ANO1* and *ANO2* have been shown to be involved in numerous diverse functions such as nociception, epithelial secretion, smooth muscle contraction, host defense, cell proliferation, and tumorigenesis [4–9], mediated by the Ca^2+^-activated Cl^−^ channel (CaCC) activity of these proteins [1, 10, 11]. Recently, it was shown that some but not all anoctamins possess CaCC activities [12]. In particular, the lack of CaCC activity for ANO3 to ANO7, is likely due to their intracellular localization [13]. Interestingly, Ano6 was found to be a CaCC [14] and a Ca^2+^-activated cation channel required for Ca^2+^-dependent phospholipid scrambling during blood coagulation [15], suggesting that different members of this family may have evolved to have different functional properties.

In 2007, it was reported in adult mouse that *Ano5* is highly expressed in skeletal muscle, cardiac muscle, and bone cells [16]. *ANO5* was the first member of this gene family reported to be associated with human diseases. Mutations in *ANO5* have been associated with gnathodiaphysial dysplasia 1(GDD1), a rare skeletal syndrome characterized by bone fragility and bony lesions of the jaw bone with autosomal dominant inheritance patterns [16–18]. Interestingly, genetic defects in *ANO5* were also identified to be responsible for two types of autosomal recessive muscular dystrophies—limb girdle muscular dystrophy type 2L (LGMD2L) and Miyoshi myopathy type 3 (MMD3) with characteristics that resemble dysferlinopathies [19–25]. Cardiac involvement was also reported to be associated with some *ANO5*-deficient patients [26–28]. Despite the clear links of *ANO5* deficiency to these genetic diseases in patients, there is currently no animal model with *ANO5* deficiency. Moreover, the cellular functions of *ANO5* in skeletal muscle and cardiac muscles remain to be determined. Therefore, we sought to determine the function of *ANO5* in these tissues by characterizing for the first time an *Ano5* knockout mouse. Our data demonstrates that complete disruption of *Ano5* expression in mice does not recapitulate the *ANO5*-deficient muscular dystrophy seen in human patients.

## Methods

### The Ano5 knockout mice

All animal studies were reviewed and approved by the Institutional Animal Care and Use Committee (IACUC) of the Ohio State University. Male C57BL/6J mice were purchased from The Jackson Laboratory (Bar Harbor, ME, USA). *Ano5* knockout mice (C57BL/6-*Ano5* < tm1Itak>) were obtained from RIKEN BioResource Center, Japan, and maintained in our barrier facility. The knockout (KO) genomic DNA was PCR amplified with a forward primer located in the upstream of the first exon of Ano5 (5′-GGGTGTTTCTGGAAGGGTGTTGT) and a reverse primer located in neomycin (5′-GTTGGCTACCCGTGATATTGCTG), or a forward primer located in neomycin (5′-GGCAGGAGCAAGGTGAGATGAC), and a reverse primer located in the intron of Ano5 (5′-GATCGCCACCTGTGCAGGCTATC), and the resulting 750 bp-product and 1100 bp-product were sequenced to define the insertion junctions. In this strain, the first exon and its upstream 1.6 kb of the *Ano5* gene was replaced with a neomycin selection cassette in the opposite orientation. The mice were backcrossed with C57BL/6J for six generations before breeding to homozygous status for the experiments. Identification of the mutant mice was performed by PCR genotyping of genomic DNA prepared from ear clips with the primers listed in Additional file 1: Table S1. The WT and KO allele would produce a 466-bp and 1200-bp band, respectively.

### RNA isolation, RT-PCR, and qRT-PCR

Total RNA extraction, reverse transcription, and PCR or quantitative PCR (qPCR) were performed as previously described [29]. In brief, total RNA was extracted from mouse tissues by using TRIzol reagent (Life Technologies, Carlsbad, CA). Total RNA was pre-treated with an DNase Ι and 5 μg of treated RNA was used as template for first-strand complementary DNA (cDNA) synthesis by using RevertAid RT Reverse Transcription Kit (Life Technologies, Carlsbad, CA). Aliquots of the RT products (50 ng) were used for regular and quantitative RT-PCR. Quantitative RT-PCR (qPCR) was performed using Radiant™ SYBR Green Hi-ROX qPCR Kits (Alkali Scientific, Pompano Beach, FL) in StepOnePlus™ Real-Time PCR Systems (Life Technologies, Carlsbad, CA) and normalized to glyceraldehyde 3-phosphate dehydrogenase (*Gapdh*). Regular RT-PCR was performed using GoTaq® Green Master (Promega, Madison, WI). The primers used in this study are listed in Additional file 1: Table S1.

### Measurement of contractile properties

The *extensor digitorum longus* (EDL) muscles were isolated and mounted as previously described [30]. Contractility assays were done at 30 °C. The optimal length of the muscle was determined using twitch contractions (single 4 ms stimulus) while stretching the muscle until maximum force was achieved. Following a 10-min rest period, the muscle underwent a single tetanic contraction (150 Hz for 250 ms). After a 5-min rest period, an eccentric contraction protocol was performed consisting of 10 tetanic contractions (150 Hz for 450 ms with a stretch equal to 3 % of optimal length for the final 200 ms) with 2 min of rest between stimulations. Twenty minutes after the tenth eccentric contraction, an 11th eccentric contraction was performed. The sutures were then removed, and the muscle was dried by placing it between a folded Kimwipe and placing a 10-g weight on top for 10 s, where after the muscle was weighed. Contractile forces are reported per unit of cross-sectional area (CSA).

### Histological analysis of frozen tissue sections

The gastrocnemius, quadriceps muscles, and heart were removed and embedded in optimal cutting temperature (OCT) compound, flash frozen using isopentane chilled in liquid nitrogen and kept at −80 °C until used. Cryosections were prepared using a cryostat Leica CM3050S. For hematoxylin and eosin (H&E) and Masson’s trichrome staining, transversely oriented sections (10 μm) were cut at mid-point and stained as previously described [31–34]. The samples were digitally imaged using a Nikon Ti-E inverted fluorescence microscope equipped with a Lumenera Infinity Color CCD camera, and a Nikon Super Fluor 20x 0.75 NA objective lens (Nikon Inc., Melville, NY, USA). The digital images were processed using the ImageJ software (NIH). The amount of fibrotic area was compared with the total area of the tissue section, and the results were expressed as a percentage of fibrotic area for each group.

### Immunohistochemistry

For immunofluorescence staining, 10-μm frozen sections were fixed with 4 % paraformaldehyde for 15 min at room temperature. The samples were then washed twice with phosphate-buffered saline (PBS) and incubated with blocking solution (PBS, 2 % BSA, 0.5 % Triton X-100, 0.1 % Tween 20, 10 % goat serum) for 1 hour before overnight incubation at 4 °C with primary antibodies (monoclonal anti-caveolin-3 antibody, C38320, BD Transduction Laboratories, 1:500; rabbit polyclonal anti-dystrophin antibody, Ab15277, Abcam, 1:200; rabbit polyclonal anti-nNOS protein antiserum H-299, Santa Cruz, 1:200; mouse monoclonal anti-dysferlin antibody, clone Ham1/7B6, Novocastra, 1:200). The slides were then extensively washed with PBS and incubated with secondary antibodies [Alexa Fluor 555 goat anti-mouse IgG (Invitrogen 1:200) or Alexa Fluor 594 goat anti-rabbit IgG (Invitrogen 1:200)] for 1 hour at room temperature. Finally, the glass slides were mounted using VECTASHIELD® Mounting Medium with 4' ,6-diamidino-2-phenylindole (DAPI) (Vector Laboratories, Inc.). Slides were analyzed using a Nikon Ti-E inverted fluorescence microscope equipped with an Andor Zyla sCMOS camera, a Nikon Super Fluor 20x 0.75 NA objective lens. Images were recorded using NIS-Elements Advanced Research software package (Nikon) and processed using Photoshop CS5 (Adobe) software package.

### Central nucleation counting

Cryosections of the gastrocnemius and quadriceps muscles were stained with anti-caveolin-3 antibody to delineate muscle fibers, and VECTASHIELD® Mounting Medium with DAPI was used for nuclear staining. All fibers, except those in direct contact with fascia, were analyzed for the location of their nuclei. For each sample group, the number of fibers with centrally localized nuclei relative to the total number of fibers was recorded. For each individual mouse, about 2500–3500 fibers were counted [33].

### Cardiotoxin-induced injury

To assess the regenerative ability after muscle damage, we induced acute muscle damage by cardiotoxin (CTX) injection (50 μl of 10 μM in sterile PBS) to the gastrocnemius muscle of *Ano5* KO mice [35]. The contralateral muscle was used as control. After 7, 14, and 21 days, the mice were sacrificed, and the muscles were dissected for histopathologic examination.

### Isoproterenol challenge and echocardiography recording

*Ano5* KO and WT mice were treated daily with intraperitoneal injections (I.P.) of 100 mg/kg isoproterenol (Sigma) for 14 days [36], and a control group was injected with an equal volume of saline. We took echocardiographic measurements before and after isoproterenol injections to assess the cardiac functions. Mice were anesthetized by isoflurane (1 %) inhalation, and echocardiographic measurements were performed with the high-resolution echocardiography analysis system for small animals (Vevo 2100TM imaging system, Visual sonics). A two-dimensional short-axis view and M-mode tracings of the left ventricle were obtained with a 30 MHz transducer. Measurements were averaged over three consecutive beats from the LV posterior wall (LVPW), the interventricular septum (IVS), and the LV internal diameter (LVID). Fractional shortening (FS) was used to estimate systolic function and was computed according to the formula FS = (LVIDd − LVIDs) / LVIDd × 100 and LV mass = 1.05 × [(LVd + IVS + PW)3 − (LVd)3], where *d* is diastole and *s* is systole.

### RNA isolation for microarray analysis

RNA was extracted by TRIzol following the manufacturer’s instructions from the gastrocnemius muscles of WT and KO mice. After RNA purification by using Quick RNA-miniPrep kit (Zymo Research, Irvine, CA), all quantitation and microarray experiments were performed at the Ohio State University Genomics Shared Resource. RNA integrity was interrogated using the Agilent 2100 Bioanalyzer (Agilent Technologies, Palo Alto, CA). A 100-ng aliquot of total RNA was linearly amplified. Then, 5.5 μg of cDNA was labeled and fragmented using the GeneChip® WT PLUS reagent kit (Affymetrix, Santa Clara, CA) following the manufacturer’s instructions. Labeled cDNA targets were hybridized to Affymetrix GeneChip® Mouse Transcriptome Array 1.0 for 16 hours at 45 °C rotating at 60 rpm. The arrays were washed and stained using the Fluidics Station 450 and scanned using the GeneChip Scanner 3000. For gene expression analysis, arrays were normalized using RMA-SST algorithm in Expression Console, and comparisons were made in Transcriptome Analysis Console (Affymetrix, Santa Clara, CA). Ingenuity pathway analysis (IPA) was used to translate the possible biological relevance of gene expression changes established (Ingenuity Systems, http://www.ingenuity.com website; Redwood City, CA, USA). Gene sets established by analysis of mRNA expression (significant expression changes) were subjected to IPA and significant pathways (*p* < 0.05) were compared with each other. Analysis settings for IPA used the reference set of Ingenuity Knowledge Base (Genes Only) with both direct and indirect relationships included. The top canonical pathways from both up- and downregulated genes were assessed for WT and KO mice.

### Western blotting

Gastrocnemius muscles from WT and *Ano5* KO mice were lysed with cold RIPA buffer supplemented with protease inhibitors, and extracted protein samples were separated by SDS-PAGE (BioRad, 4–20 %) and transferred onto PVDF membranes (0.45 μm). Primary antibodies include the rabbit polyclonal anti-dystrophin (E2660, 1:500, Spring Bioscience, Pleasanton, CA), anti-nNOS (H-299,1:3000, SantaCruz Biotechnology, SantaCruz, CA, USA), anti-ANO1 antibody (53213, 1:1, Abcam, Cambridge, MA), mouse monoclonal anti-dysferlin antibody (NCL-Hamlet, Ham1/7B6,1:500, Novocastra, Newcastle, UK), anti-ANO6 antibody (N429/19, 1:40, UC Davis/NIH NeuroMab Facility, UC Davis, USA), and anti-Gapdh (MAB374, 1:2500, Millipore, Billerica, MA). Secondary antibodies were HRP-conjugated rabbit anti-mouse (1:3000, and goat anti-rabbit secondary antibodies (1:3000) (Millipore, Billerica, MA). The membranes were developed using ECL Western blotting substrate (Pierce Biotechnology, Rockford, IL) and exposed to film (Kodak, Rochester).

*Statistical analysis—*Data are expressed as mean ± standard deviation (SD). Statistical differences were determined by unpaired Student’s *t* test for two groups and one-way ANOVA with Bonferroni’s post tests for multiple group comparisons using Prism 5.02 (Graphpad Software, La Jolla, California). A *p* value less than 0.05 was considered to be significant.

## Results

### Expression of Ano5 in different mouse tissues

**Fig. 1:**
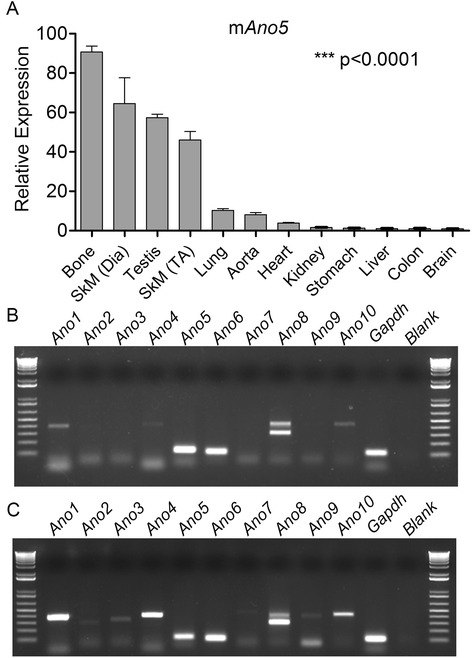
Expression of *Ano5* in different mouse tissues and expression of different anoctamins in mouse skeletal muscle and heart. **a** Expression of *Ano5* in different mouse tissues examined by quantitative RT-PCR. SkM (Dia), skeletal muscle (diaphragm); SkM (TA), skeletal muscle (tibialis anterior). Four mice of each group were used. **b**, **c** Expression of different anoctamin genes in the skeletal muscle (**b**) and heart (**c**) by regular RT-PCR. These data are representative of at least four samples

We first examined the expression of *Ano5* in various mouse tissues including skeletal muscle and heart by quantitative RT-PCR. *Ano5* was found to be highly expressed in the bone, skeletal muscle and testis, moderately in the lung and aorta, mildly in the heart, and low in the kidney, stomach, liver, colon, and brain (Fig. 1a). In addition, we examined the expression of various anoctamin genes in mouse skeletal muscle and heart. As shown in Fig. 1b, *Ano5*, *6*, and *8* were expressed relatively high in skeletal muscle, followed by *Ano1*, *4*, and *10*, while *Ano2*, *3*, *7*, and *9* were hardly detectable. In mouse heart, *Ano1*, *4*, *5*, *6*, and *8* were expressed relatively high, followed by *Ano3* and *9*, while *Ano2* and *Ano7* bands were only faintly visible (Fig. 1c).

### Generation of the Ano5 knockout mice

**Fig. 2:**
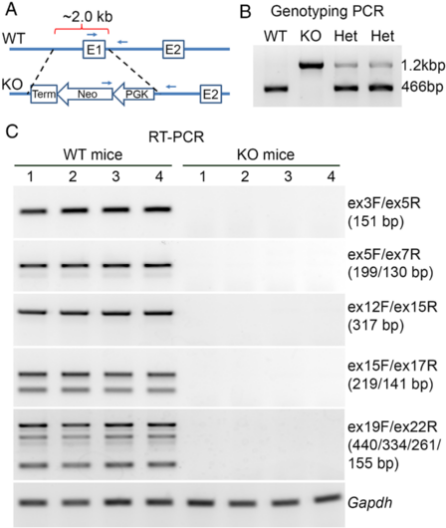
Generation of *Ano5* KO mice. **a** Schematic representation of WT and KO alleles of mouse *Ano5*. A neomycin cassette was inserted in the exon 1 of mouse *Ano5*. **b** Genotyping of the WT, KO, and heterozygous (Het) mice. **c** RT-PCR analysis of the skeletal muscles from WT and KO mice (four in each group) using four different primer sets. *Gapdh* was used as a reference gene

To disrupt the expression of *Ano5* in mice, the first exon of *Ano5* and its upstream about 1.6 kb were replaced with a neomycin resistance cassette in the opposite orientation (Fig. 2a). Genotyping PCR analysis revealed the presence of the KO allele in the heterozygous and homozygous mutant mice as expected (Fig. 2b). The WT allele was not detected in the homozygous mutant mice (Fig. 2b). Breeding the heterozygous male and female mice yielded the expected ~1:2:1 ratio of three genotypes (WT, heterozygous, and homozygous KO). Due to the unavailability of a well-characterized Ano5 antibody, we employed RT-PCR to examine whether replacement of the first exon and its upstream region with the neomycin cassette disrupted the expression of *Ano5* in the skeletal muscle of mice. Five different primer sets faithfully amplified different regions along the *Ano5* transcripts (including different alternatively spliced variants) from the WT skeletal muscle, but there were no PCR products from the KO skeletal muscle (Fig. 2c). We have tested several commercial anti-Ano5 antibodies, but unfortunately, they did not work to detect Ano5 by Western blotting (Additional file 2: Figure S1). However, our RT-PCR data convincingly demonstrate that the KO mice completely lack *Ano5* expression and thus are suitable for studying the physiological consequence of *Ano5* deficiency.

### Normal contractile responses in Ano5-KO muscles

**Fig. 3:**
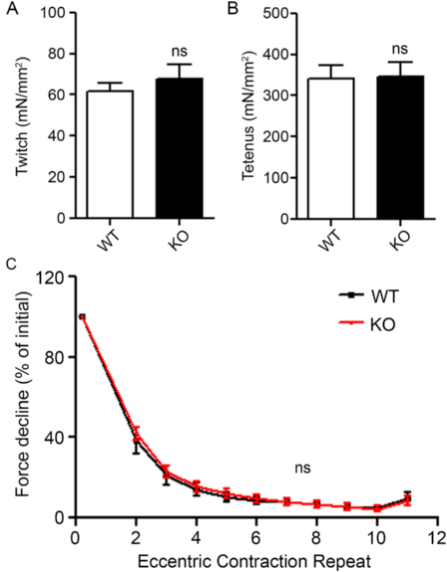
Measurement of the contractile properties in the EDL muscles. Specific force production of both twitch (**a**) and tetanic contractions at 150Hz for 250 ms (**b**) was not different between WT and KO muscles. **c** Tetanic force declines with repeated eccentric contractions. Contractile forces are reported per unit of cross-sectional area (CSA). *N* of mice = 6 and 7 for KO and WT at 4 months of age. *ns*, no statistical significance

Two important features of muscular dystrophy are that the muscle produces reduced force and it is more susceptible to lengthening-contraction-induced (LC-induced) damage. We thus examined the effect of *Ano5* knockout on force production and force deficit in response to LC-induced muscle injury by measuring the ex vivo contractile properties of the *extensor digitorum longus* (EDL) muscles [37]. WT and KO muscles showed no statistically significant differences in maximal force when subjected to twitch contractions (67.7 ± 6.7 mN/mm^2^, *n* = 6 and 75.1 ± 14.1 mN/mm^2^, *n* = 7 for KO and WT, respectively; *p* = 0.66) (Fig. 3a) or tetanic contraction at 150 Hz (345.7 ± 36.8 mN/mm^2^, *n* = 6 and 374.8 ± 42.5 mN/mm^2^, *n* = 7 for KO and WT, respectively; *p* = 0.62) (Fig. 3b). The EDL muscle was then subjected to a train of 10 lengthening contractions with 2 min of rest between stimulations (see “Methods” section for details), and the force deficit in the KO EDL muscle was indistinguishable from that in the WT control at 4 months of age (Fig. 3c). In summary, the contractile properties of the *Ano5* KO muscles were not significantly different from the WT muscles.

### No overt muscle pathology in the KO mice

**Fig. 4:**
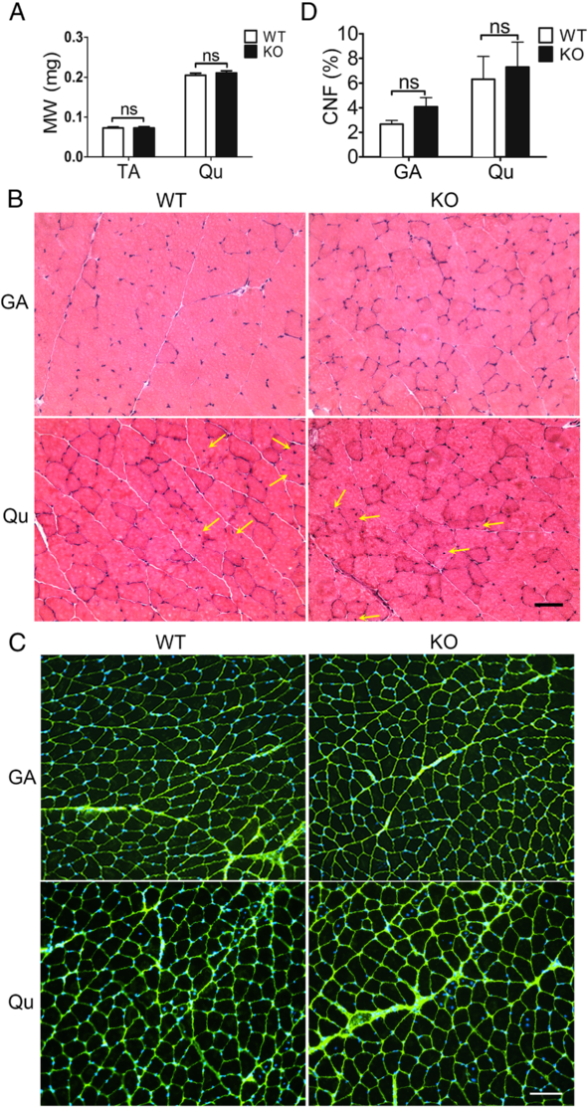
Characterization of the muscular phenotype in the *Ano5* KO mice. **a** *Tibialis anterior* (*TA*) and q*uadriceps* (*Qu*) muscle mass (*MM*) normalized to body weight (*BW*) from the indicated genotypes of mice. **b** H&E-stained cryosections of the *gastrocnemius* (*GA*) and quadriceps (*Qu*) from 18-month-old mice of the indicated genotypes. *Scale bar* = 50 μm. **c** Representative immunofluorescence images for caveolin-3 (*green*) and nuclei (DAPI, *blue*) staining in gastrocnemius and quadriceps sections from 18-month-old WT and KO mice. *Scale bar* = 100 μm. **d** Percentage of myofibers with centrally located nuclei from the gastrocnemius and quadriceps muscles of WT and KO mice at 18 months of age. *ns*, no statistical significance. *N* of mice: 6–8 for each group

Recessive mutations in *ANO5* have been shown to cause LGMD2L and MMD3 in a wide range of clinical patients [25]. In order to study the effect of *ANO5* deficiency on skeletal muscle, we sought to examine the *Ano5* KO mice for any signs of muscular dystrophy. Our initial gross and histological examination of the young KO mice did not reveal any obvious pathology as compared to their WT littermate controls (data not shown). We further analyzed the mice at older ages (8 and 18 months) by histopathological analysis. The muscle mass normalized to body weight of both the tibialis anterior and gastrocnemius muscles were not significantly changed in the KO mice as compared to their WT littermates (Fig. 4a). H&E staining of muscle sections showed no major histopathological alterations in the gastrocnemius and quadriceps muscles of the KO mice compared with their age-matched WT littermates (Fig. 4b and Additional file 3: Figure S2). There was no increase in fibrosis in the KO muscles as studied with the Masson’s trichrome staining (Additional file 4: Figure S3). Analysis of myofiber central nucleation by co-labeling the muscle fibers with caveolin-3 and DAPI (Fig. 4c) showed a small age-related increase in the quadriceps muscles at 18 months of age in both WT and KO mice. However, no significant changes between these two groups were identified (Fig. 4c, d). Furthermore, no changes in regenerative ability were noted between WT and KO at 7, 14, and 21 days after an acute injury with cardiotoxin (Additional file 5: Figure S4). Our data suggest that disruption of *Ano5* expression in mice does not cause overt muscle histopathology up to 18 months of age under resting conditions.

### Intact DGC and dysferlin in the absence of Ano5

**Fig. 5:**
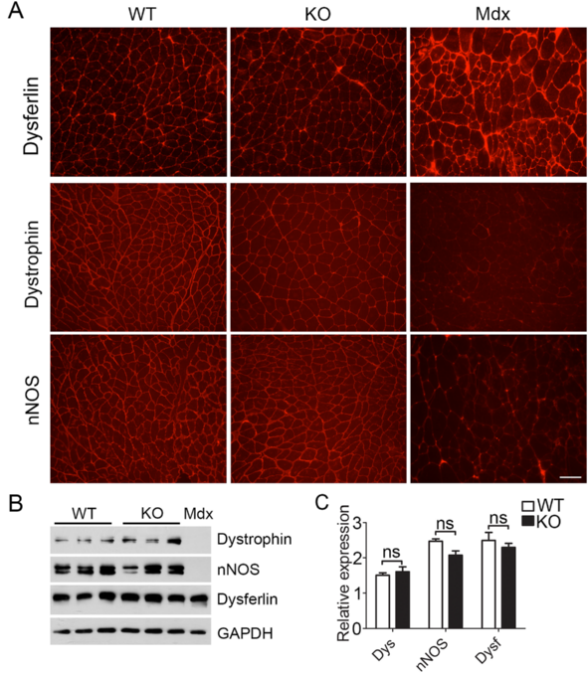
The expression of DGC and dysferlin in *Ano5* KO mice. **a** Representative immunofluorescence images for Dysferlin, Dystrophin and nNOS staining in gastrocnemius sections from 18-month-old WT mice and *Ano5* KO mice. **b**, **c** Representative images and quantification of DGC and dysferlin expression by Western blotting from the gastrocnemius of 18-month-old WT mice and *Ano5* KO mice. Dys, Dystrophin; Dysf, Dysferlin; nNOS, neuronal nitric oxide synthase. *Scale bar* = 100 μm. ∗∗∗*p* < 0.01 versus WT controls. ns, no statistical significance. The number of mice is 6–8 for each group

Disruption of membrane stability due to the loss of dystrophin-glycoprotein complex (DGC) or membrane repair capacity due to the defect in dysferlin has been widely reported to be responsible for various types of muscular dystrophies [34, 38, 39]. To examine whether Ano5 deficiency may alter the expression and/or localization of DGC and dysferlin, we performed immunofluorescence staining and Western blotting analyses on the skeletal muscles from WT and KO mice. The DGC components such as dystrophin and nNOS and membrane repair protein dysferlin were all normally expressed and localized at the sarcolemma of the *Ano5*-KO skeletal muscle (Fig. 5a-c).

### Normal cardiac function in Ano5 KO mice

**Fig. 6:**
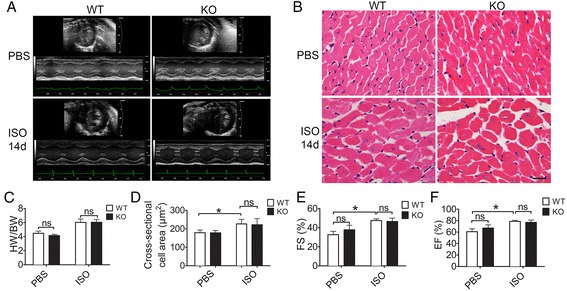
Normal cardiac phenotype in 18-month-old *Ano5* KO mice after I.P. injection of 100 mg/kg ISO for 14 days. **a** Representative serial M-mode echocardiography in WT and *Ano5* KO mice measured before and 14 days after ISO treatment. **b** H&E-stained cross sections of WT and *Ano5* KO mice hearts subjected to PBS or ISO for 2 weeks. **c** The ratio of heart/body weight in WT and *Ano5* KO mice measured before and 14 days after ISO treatment. **d** Quantification of mean cross-sectional area of cardiomyocyte from WT and *Ano5* KO mice. (**e**, **f**) Graph representing the mean fractional shortening (FS) and ejection fraction (EF) as measured by M-mode echocardiography in WT and *Ano5* KO mice before and 14 days after ISO treatment. *Scale bar* = 20 μm. ∗*p* < 0.05 versus WT controls. *ns*, no statistical significance. The number of mice is 5–8 for each group

Previous studies reported the expression of *ANO5* in both skeletal and cardiac tissues in humans [17]. Recent studies on the cardiac conduit system in *ANO5* mutant patients also suggested an increased risk of ventricular arrhythmia [26, 40]. We therefore compared the heart function of the *Ano5* KO mice and their WT littermates under resting conditions or with isoproterenol (ISO)-induced left ventricular hypertrophy at 18 months of age. Our data showed no significant differences in the echocardiography images (Fig. 6a), histology (Fig. 6b), heart/body weight ratio (Fig. 6c), the cell size of the left ventricle (Fig. 6b, d), or fibrosis (Additional file 6: Figure S5) between the KO and WT groups at baseline. The cardiac functions (fractional shortening and ejection fraction) as measured by echocardiography were also normal in the KO mice (Fig. 6e, f).

To further test if ISO-induced cardiac stress can unmask any cardiac defect in the *Ano5* KO mice, we studied the cardiac function by echocardiography in the mice treated with daily ISO (100 mg/kg, I.P.) injections for 14 days. Both *Ano5* KO and WT hearts developed significant left ventricular dilation after ISO injections (Fig. 6a and Additional file 7: Table S2). However, no differences were discernible in the thickness of the interventricular septum between *Ano5* KO and WT groups over the course of the experiments (Additional file 7: Table S2). The percentage increase in myocyte transverse cross-sectional area showed no significant differences between the KO and WT mice (Fig. 6c, d). By 2 weeks, neither fractional shortening (WT, 47.54 ± 1.81 %, *n* = 5; *Ano5* KO, 46.51 ± 3.60 %, *n* = 5; *p* = 0.84) nor ejection fraction (WT, 78.79 ± 1.86 %, *n* = 5; *Ano5* KO, 77.08 ± 4.04 %, *n* = 5; *p* = 0.77) was significantly different between these two groups.

### Analysis of other anoctamin genes in Ano5 KO mice

**Fig. 7:**
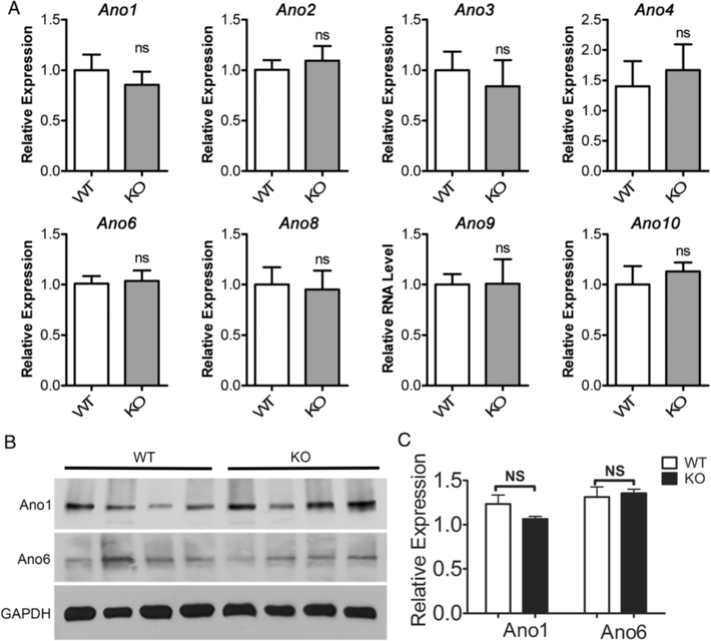
**a** Expression of different anoctamin genes in WT and KO skeletal muscles by quantitative RT-PCR. Four mice at 18 months of age in each group were used. Note: *Ano7* was not detectable in mouse skeletal muscle. **b**, **c** Representative images and quantification of *Ano1* and *Ano6* expression by Western blotting from the gastrocnemius of 18-month-old WT mice and *Ano5* KO mice. Four mice in each group were used

There are a total of ten members of the anoctamin family with some overlapping expression in each tissue such as skeletal muscle (Fig. 1b). To test whether the lack of muscular dystrophy phenotype in the *Ano5* KO mice might be due to the upregulation of other anoctamin genes, we compared the expression of all nine other anoctamin genes in skeletal muscle between WT and KO mice by quantitative RT-PCR. Strikingly, none of these genes showed significant changes between these two genotypes (Fig. 7a). These data suggest that transcriptional upregulation of other anoctamins is not responsible for the lack of phenotypic manifestations in *Ano5* KO mice. To further test whether the expression of some anoctamins at the protein level is altered in *Ano5* KO skeletal muscle, we performed Western blotting analysis and our data showed that there was no significant difference in *Ano1* and *Ano6* protein expression between WT and KO skeletal muscles (Fig. 7b, c).

### Microarray analysis

To determine the general picture of gene expression alterations in *Ano5* KO skeletal muscles, we performed microarray analysis of the total RNAs of the gastrocnemius muscles from WT and KO mice (*n* = 3 per genotype). Consistent with the RT-PCR results (Fig. 2c), microarray analysis showed that *Ano5* is the most downregulated gene (with an over 96-fold change) (Table 1), further, confirming our *Ano5* KO mice is truly deficient in the expression of *Ano5*. Transcripts that were calculated to have log2 ratio intensity between KO and WT muscles greater than 0.8 or lower than −0.8 were recognized as upregulated and downregulated, respectively. A total number of 65,956 genes were screened, among which, 81 genes were upregulated (Additional file 8: Table S3) and 893 genes downregulated (Additional file 9: Table S4). Specifically, albumin (*Alb*), resistin (*Retn*), and stearoyl-CoA desaturase (*Scd*), key enzymes in fatty acid metabolism, were all downregulated in KO muscles by more than 30-, 27-, and 22-fold, respectively. Moreover, Complement factor D (Cfd) was more than 10-fold downregulated, and haptoglobin (*Hp*) and transferrin (*Tf*) were also downregulated by more than 11- and 9-fold. Key upregulated molecules were Kelch-like protein 33 (*Klhl33*) and peptidase M20 domain-containing protein 2 (*Pm20d2*) that is an important molecule in metabolism. Coenzyme Q5 (*Coq5*), branched chain ketoacid dehydrogenase kinase, and nitric oxide synthase 1 (*Nos1*) were all upregulated (Table 1).

**Table 1:** Top upregulated and downregulated genes in Ano5 KO skeletal muscles by microarray analysis

Gene	Full name	Fold change
*Klhl33*	Kelch-like protein 33	↑ 4.440
*Pm20d2*	Peptidase M20 domain-containing protein 2	↑ 3.260
*Ube3b*	Ubiquitin protein ligase E3B	↑ 2.980
*Dennd4b*	DENN domain-containing protein 4B	↑ 2.870
*C10orf71*	Chromosome 10 open reading frame 71	↑ 2.660
*Coq5*	Coenzyme Q5	↑ 2.570
*Bckdk*	Branched chain ketoacid dehydrogenase kinase	↑ 2.440
*Nos1*	Nitric oxide synthase 1	↑ 2.410
*Rapsn*	Receptor-associated protein of the synapse	↑ 2.410
*Sim2*	Single-minded family BHLH transcription factor 2	↑ 2.410
*Ano5*	Anoctamin 5	↓−96.640
*Alb*	Albumin	↓−30.130
*Retn*	Resistin	↓−27.420
*Scd*	Stearoyl-coA desaturase	↓−22.820
*Cdo1*	Cysteine dioxygenase type 1	↓−15.030
*Hp*	Haptoglobin	↓−11.890
*Cfd*	Complement factor D	↓−11.520
*Tf*	Transferrin	↓−9.990

**Fig. 8:**
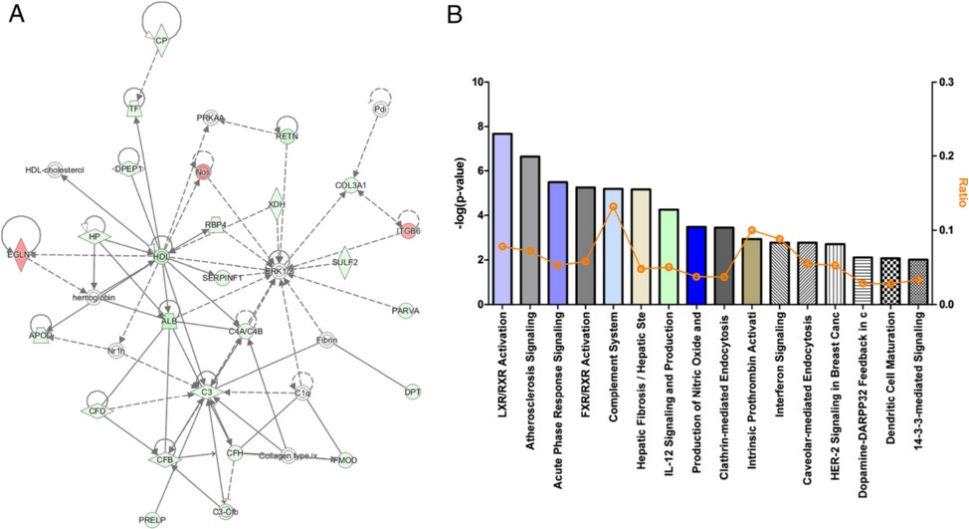
Ingenuity pathway analysis (IPA) of the microarray data from the gastrocnemius muscles from WT and KO mice. **a** Genes involved in the high-density lipoprotein synthesis pathway were altered in Ano5 KO skeletal muscles. *Light green*: downregulated; *Red*: upregulated. **b** Top canonical pathways suggested by IPA showed lipid metabolism and complement pathways are altered in Ano5 KO skeletal muscles. Number of mice per group is 3

Ingenuity pathway analyses (IPA) performed on gene expression array data from muscles of WT and KO mice showed general changes in lipid metabolism and adiposity pathways. In particular, expression of *Alb*, *Hp*, *Tf*, *Cfd*, *Retn*, and *Nos1* genes in the high-density lipoprotein (HDL) synthesis pathway (Fig. 8a) was significantly altered in the *Ano5* KO muscle. Moreover, top canonical pathways suggested by IPA include lipid X receptor and retinoid X receptor (LXR/RXR) and farnesoid X receptor and retinoid X receptor (FXR/RXR) activation pathways (Fig. 8b). The LXR/RXR activation pathway is involved in the regulation of lipid metabolism, inflammation, and cholesterol [41]. The FXR/RXR activation pathway plays a crucial role in lipoprotein, lipid, and glucose metabolism and has emerged as a general key player in the control of numerous metabolic pathways [42]. Moreover, the complement system was also significantly affected in the KO skeletal muscles. In summary, microarray data and IPA analyses suggest changes in metabolic and complement pathways in *Ano5* KO skeletal muscles.

## Discussion

*ANO5* is highly expressed in human skeletal muscle, cardiac muscle, chondrocytes, and osteoblasts [16], and it was the first member of the anoctamin family reported to be associated with human diseases. Genetic defects in *ANO5* were identified to be responsible for two types of autosomal recessive muscular dystrophies [19, 20, 25, 26, 40]. Furthermore, cardiac involvement was also reported in association with some *ANO5*-deficient patients [26, 28]. All these clinical studies support an essential role of *ANO5* in the human musculoskeletal and cardiac systems. However, our present work using a reverse genetic approach demonstrates that *Ano5* is dispensable in the striated muscles of mice, despite the high expression of *Ano5* in the mouse skeletal muscles. The *Ano5* KO allele lacks the first exon of Ano5 and its upstream region of about 1.6 kb, within which the promoter of the *Ano5* gene is likely located. Consistently, the *Ano5* transcripts were completely gone in the *Ano5* KO mice, indicating these are authentic *Ano5* KO mice.

The *Ano5* KO mice did not display any obvious signs of muscular dystrophy as examined by various established methods. The muscle mass was not affected at 8 and 18 months in the absence of *Ano5*. The musculature was essentially indistinguishable between the KO and WT mice. Analysis of myofiber central nucleation showed a small, non-statistical increase in the quadriceps muscles up to 18 months of age in both WT and KO mice. Disruption of the membrane stability or the membrane repair capacity has been widely reported to be responsible for various types of muscular dystrophies [34, 38, 39]. However, the expression and localization of DGC components and dysferlin were unaltered in the KO mice. Moreover, no changes in regenerative ability were noted between WT and KO mice after an acute injury with cardiotoxin. In addition, the KO mice showed normal cardiac phenotype when compared to WT controls with no major differences in the thickness of the interventricular septum or cardiac functions. Following ISO challenge for 2 weeks, both KO and WT hearts developed left ventricular dilation similarly without significant difference between the two groups.

The lack of muscular dystrophy phenotype in our *Ano5* KO mice is in sharp contrast to what has been reported in clinical studies. These results may cast uncertainty on the pathogenic role of human *ANO5* mutations in muscular dystrophy. However, this is less likely for the following reasons. First, *ANO5* was shown to be highly expressed in human skeletal and cardiac tissues [16]. Second, genetic mapping studies linked the *ANO5* region to be responsible for LGMD2L and MMD3 patients, and mutations in *ANO5* were also found in some of these patients [25, 43]. Third, muscular dystrophy with mutations in *ANO5* appears to be one of the most common adult muscular dystrophies in Northern Europe [19, 40]. Fourth, the pathogenicity of the *ANO5* mutations is supported by their recurrence in multiple unrelated patients and their segregation in affected family members [40].

The difference in mutation types might cause different phenotypic consequences. Previous clinical studies reported various mutation types including missense, frameshift, splice site, and nonsense mutations of *ANO5* in the LGMD2L and MMD3 patients [19, 25, 40]. Such mutations may result in the expression of truncated or mutated versions of *ANO5*, which could be pathogenic. However, our study employed complete disruption of *Ano5* transcription via insertion of a neomycin cassette into the first exon of *Ano5*. These KO mice would be spared from such pathogenic peptides and thus little phenotype would be presented. However, this possibility is low because the heterozygous parents of the *ANO5*-mutant patients were not reported to have the disease. Interestingly, dominant mutations of *ANO5* were reported to cause GDD1 in humans [17], but so far, only recessive mutations in *ANO5* were reported to cause muscular dystrophy. It is unclear whether the GDD1 patients display the muscular dystrophy phenotype or vice versa.

A more plausible explanation for the different results of *Ano5* deficiency between our study and the clinical findings is the species difference. It is crucial to note the possibility that any given response in a mouse may not occur in exactly the same pattern in humans [44], as many changes need to accumulate before being expressed and mice are much smaller and live significantly shorter than humans. Moreover, mice are different from humans in various aspects such as immunity [44], metabolism [45], and genetics [46], and living environment. Therefore, it is possible that other factors (e.g., genetic, environmental, metabolic, and/or immunological), which are most likely present in humans but not (or to a lesser extent) in mice, are involved in the pathogenic role of *ANO5* mutation. It is also possible that additional factors, which are most likely present in mice but not (or to a lesser extent) in humans, play a protective or compensatory role to prevent the pathogenic consequence of *Ano5* deficiency. It has previously been noticed that the differences in the compensatory pathways between mice and humans can lead to different phenotype severity of muscular dystrophy between these two species. For example, utrophin is well known to compensate for the loss of dystrophin in mice and results in a much milder phenotype than in human Duchenne muscular dystrophy [47, 48]. Identifying such compensatory mechanisms could potentially lead to the development of novel therapies for *ANO5*-deficient muscular dystrophy. Interestingly, there are ten different members of the anoctamin family with significant sequence homology and some overlapping expression in a given tissue. It is thus possible that the loss of *Ano5* in mice is compensated by other anoctamin proteins expressed. Our RT-PCR data showed that *Ano6* and *8* were also expressed in skeletal muscle besides *Ano5*. However, none of these or other anoctamin genes were expressed differently in *Ano5* KO skeletal muscles, suggesting that it is less likely that other anoctamins compensate for the loss of *Ano5* in mice, at least at the transcriptional level. To fully exclude such possibility, future studies would need to be carried out using double or triple knockout approaches.

Interestingly, although the *Ano5* KO mice did not exhibit overt muscle pathology, our microarray analysis showed that some important signaling pathways were dysregulated in the KO muscle. In particular, expression of the genes involved in the lipid metabolism and innate immune pathways were significantly altered. These data suggest a potential function of Ano5 in maintaining the metabolic homeostasis in the skeletal muscle.

## Conclusions

The results presented in this study demonstrates that genetic ablation of *Ano5* in C57BL6/J mice does not recapitulate *ANO5*-deficient muscular dystrophy and associated cardiomyopathy as in human patients. However, Ano5 deficiency results in altered gene expression in the lipid metabolism and complement signaling pathways.

## Additional files

Additional file 1: Table S1. Primer list. Primers for genotyping, RT, and real-time PCR. Additional file 2: Figure S1. Test of anti-Ano5 antibodies by Western blotting. a The anti-Ano5 antibody (sc-169626) from Santa Cruz Biotechnology did not detect any positive signal in human Ano5-expressing cell lysate, which was correctly detected by the anti-GFP antibody at the predicted size. b The anti-Ano5 antibody (AP8580B) from Abgent produced a prominent band at about 100 kDa in both negative and positive samples; however, this band is not Ano5 because the Ano5-YFP fusion protein should be around 135 kDa as correctly detected by the anti-GFP antibody. The *arrows* point to the correct Ano5-YFP fusion protein. These experiments were repeated at least three times. Additional file 3: Figure S2. H&E-stained histological sections of the gastrocnemius and quadriceps from 8-month-old mice of the indicated genotypes. *Scale bar* = 50 μm. The number of mice is 6–8 for each group. Additional file 4: Figure S3. Masson’s trichrome-stained histological sections of the gastrocnemius and quadriceps from 8- to 18-month-old mice of the indicated genotypes. *Scale bar* = 50 μm. The number of mice is 6–8 for each group. Additional file 5: Figure S4. Muscle regeneration after cardiotoxin injury in *Ano5* KO mice. a Representative H&E-stained gastrocnemius muscle sections from WT or *Ano5* KO mice after 7, 14, and 21 days post-CTX-induced injury. *Scale bar* = 100 μm. b Representative immunofluorescence images for caveolin-3 (*green*) and nucleus (DAPI, *blue*) staining in gastrocnemius muscle sections from WT and *Ano5* KO mice at 14 days after CTX injection. *Scale bar* = 50 μm. c Quantitative analysis of the cross-sectional area of muscle fibers of the WT and KO mice at day 14 post-injury. *ns*, no statistical significance. *N* of mice: 3–5 for each group. Additional file 6: Figure S5. Representative images of Masson’s trichrome-stained histological sections of WT and *Ano5* KO hearts after PBS or ISO treatments for 2 weeks. *Scale bar* = 50 μm. The number of mice (18 months of age) is 6–8 for each group. Additional file 7: Table S2. Echocardiographic measurements. Echocardiographic measurements in WT and Ano5 KO mice after isoproterenol injections. Additional file 8: Table S3. Gene list. List of genes that are downregulated in the KO skeletal muscles. Additional file 9: Table S4. Gene list. List of genes that are upregulated in the KO skeletal muscles.

## Abbreviations

Ano5: anoctamin 5
CaCC: Ca^2+^-activated chloride channel
CK: creatine kinase
DGC: dystrophin-glycoprotein complex
GDD1: gnathodiaphyseal dysplasia 1
ISO: isoproterenol
KO: knockout
LGMD2L: limb girdle muscular dystrophy 2L
MMD3: Miyoshi myopathy type 3
WT: wild type

## References

[1] Yang YD, Cho H, Koo JY, Tak MH, Cho Y, Shim WS (2008). TMEM16A confers receptor-activated calcium-dependent chloride conductance. Nature.

[2] Duran C, Hartzell HC (2011). Physiological roles and diseases of Tmem16/Anoctamin proteins: are they all chloride channels?. Acta Pharmacol Sin.

[3] Park SH, Chung HK, Kim do J, Han MR, Park MS, Oh U (2011). Overexpression, crystallization and preliminary X-ray crystallographic analysis of the C-terminal cytosolic domain of mouse anoctamin 1. Acta Crystallogr Sect F: Struct Biol Cryst Commun.

[4] Manoury B, Tamuleviciute A, Tammaro P (2010). TMEM16A/anoctamin 1 protein mediates calcium-activated chloride currents in pulmonary arterial smooth muscle cells. J Physiol.

[5] Duvvuri U, Shiwarski DJ, Xiao D, Bertrand C, Huang X, Edinger RS (2012). TMEM16A induces MAPK and contributes directly to tumorigenesis and cancer progression. Cancer Res.

[6] Huang F, Zhang H, Wu M, Yang H, Kudo M, Peters CJ (2012). Calcium-activated chloride channel TMEM16A modulates mucin secretion and airway smooth muscle contraction. Proc Natl Acad Sci U S A.

[7] Liu W, Lu M, Liu B, Huang Y, Wang K (2012). Inhibition of Ca(2+)-activated Cl(−) channel ANO1/TMEM16A expression suppresses tumor growth and invasiveness in human prostate carcinoma. Cancer Lett.

[8] Wong XM, Younger S, Peters CJ, Jan YN, Jan LY (2013). Subdued, a TMEM16 family Ca(2)(+)-activated Cl(−)channel in Drosophila melanogaster with an unexpected role in host defense. Elife.

[9] Britschgi A, Bill A, Brinkhaus H, Rothwell C, Clay I, Duss S (2013). Calcium-activated chloride channel ANO1 promotes breast cancer progression by activating EGFR and CAMK signaling. Proc Natl Acad Sci U S A.

[10] Caputo A, Caci E, Ferrera L, Pedemonte N, Barsanti C, Sondo E (2008). TMEM16A, a membrane protein associated with calcium-dependent chloride channel activity. Science.

[11] Terashima H, Picollo A, Accardi A (2013). Purified TMEM16A is sufficient to form Ca2+-activated Cl-channels. Proc Natl Acad Sci U S A.

[12] Schreiber R, Uliyakina I, Kongsuphol P, Warth R, Mirza M, Martins JR (2010). Expression and function of epithelial anoctamins. J Biol Chem.

[13] Duran C, Qu ZQ, Osunkoya AO, Cui YY, Hartzell HC (2012). ANOs 3-7 in the anoctamin/Tmem16 Cl-channel family are intracellular proteins. Am J Physiol-Cell Ph.

[14] Shimizu T, Iehara T, Sato K, Fujii T, Sakai H, Okada Y (2013). TMEM16F is a component of a Ca2+-activated Cl-channel but not a volume-sensitive outwardly rectifying Cl-channel. Am J Physiol-Cell Ph.

[15] Yang H, Kim A, David T, Palmer D, Jin T, Tien J (2012). TMEM16F forms a Ca2+-activated cation channel required for lipid scrambling in platelets during blood coagulation. Cell.

[16] Mizuta K, Tsutsumi S, Inoue H, Sakamoto Y, Miyatake K, Miyawaki K (2007). Molecular characterization of GDD1/TMEM16E, the gene product responsible for autosomal dominant gnathodiaphyseal dysplasia. Biochem Biophys Res Commun.

[17] Tsutsumi S, Kamata N, Vokes TJ, Maruoka Y, Nakakuki K, Enomoto S (2004). The novel gene encoding a putative transmembrane protein is mutated in gnathodiaphyseal dysplasia (GDD). Am J Hum Genet.

[18] Marconi C, Binello PB, Badiali G, Caci E, Cusano R, Garibaldi J (2013). A novel missense mutation in ANO5/TMEM16E is causative for gnathodiaphyseal dyplasia in a large Italian pedigree. Eur J Hum Genet.

[19] Hicks D, Sarkozy A, Muelas N, Koehler K, Huebner A, Hudson G (2011). A founder mutation in Anoctamin 5 is a major cause of limb-girdle muscular dystrophy. Brain.

[20] Little AA, McKeever PE, Gruis KL (2013). Novel mutations in the Anoctamin 5 gene (ANO5) associated with limb-girdle muscular dystrophy 2L. Muscle Nerve.

[21] Magri F, Del Bo R, D’Angelo MG, Sciacco M, Gandossini S, Govoni A (2012). Frequency and characterisation of anoctamin 5 mutations in a cohort of Italian limb-girdle muscular dystrophy patients. Neuromuscul Disord.

[22] Penisson-Besnier I, Saint-Andre JP, Hicks D, Sarkozy A, Croue A, Hudson J (2012). Myopathy caused by anoctamin 5 mutations and necrotizing vasculitis. J Neurol.

[23] Schessl J, Kress W, Schoser B (2012). Novel ANO5 mutations causing hyper-CK-emia, limb girdle muscular weakness and Miyoshi type of muscular dystrophy. Muscle Nerve.

[24] Witting N, Duno M, Petri H, Krag T, Bundgaard H, Kober L (2013). Anoctamin 5 muscular dystrophy in Denmark: prevalence, genotypes, phenotypes, cardiac findings, and muscle protein expression. J Neurol.

[25] Bolduc V, Marlow G, Boycott KM, Saleki K, Inoue H, Kroon J (2010). Recessive mutations in the putative calcium-activated chloride channel Anoctamin 5 cause proximal LGMD2L and distal MMD3 muscular dystrophies. Am J Hum Genet.

[26] Witting N, Duno M, Petri H, Krag T, Bundgaard H, Kober L (2013). Anoctamin 5 muscular dystrophy in Denmark: prevalence, genotypes, phenotypes, cardiac findings, and muscle protein expression. J Neurol.

[27] Liewluck T, Winder TL, Dimberg EL, Crum BA, Heppelmann CJ, Wang Y (2013). ANO5-muscular dystrophy: clinical, pathological and molecular findings. Eur J Neurol.

[28] Wahbi K, Behin A, Becane HM, Leturcq F, Cossee M, Laforet P (2013). Dilated cardiomyopathy in patients with mutations in anoctamin 5. Int J Cardiol.

[29] Zhao P, Torcaso A, Mariano A, Xu L, Mohsin S, Zhao L et al. Anoctamin 6 regulates C2C12 myoblast proliferation. PloS One. 9(3):e92749. doi:10.1371/journal.pone.0092749. 10.1371/journal.pone.0092749 PMC3963950 24663380

[30] Janssen PM, Murray JD, Schill KE, Rastogi N, Schultz EJ, Tran T (2014). Prednisolone attenuates improvement of cardiac and skeletal contractile function and histopathology by lisinopril and spironolactone in the mdx mouse model of Duchenne muscular dystrophy. PLoS One.

[31] Han R, Rader EP, Levy JR, Bansal D, Campbell KP (2011). Dystrophin deficiency exacerbates skeletal muscle pathology in dysferlin-null mice. Skelet Muscle.

[32] Zhao P, Xu L, Ait-Mou Y, de Tombe PP, Han R (2011). Equal force recovery in dysferlin-deficient and wild-type muscles following saponin exposure. J Biomed Biotechnol.

[33] Han R, Frett EM, Levy JR, Rader EP, Lueck JD, Bansal D (2010). Genetic ablation of complement C3 attenuates muscle pathology in dysferlin-deficient mice. J Clin Invest.

[34] Han R, Kanagawa M, Yoshida-Moriguchi T, Rader EP, Ng RA, Michele DE (2009). Basal lamina strengthens cell membrane integrity via the laminin G domain-binding motif of alpha-dystroglycan. Proc Natl Acad Sci U S A.

[35] d’Albis A, Couteaux R, Janmot C, Roulet A, Mira JC (1988). Regeneration after cardiotoxin injury of innervated and denervated slow and fast muscles of mammals. Myosin isoform analysis. Eur J Biochem.

[36] Zhang R, Khoo MSC, Wu YJ, Yang YB, Grueter CE, Ni GM (2005). Calmodulin kinase II inhibition protects against structural heart disease. Nat Med.

[37] Brooks SV, Faulkner JA (1988). Contractile properties of skeletal muscles from young, adult and aged mice. J Physiol.

[38] Bansal D, Miyake K, Vogel SS, Groh S, Chen CC, Williamson R (2003). Defective membrane repair in dysferlin-deficient muscular dystrophy. Nature.

[39] Cohn RD, Campbell KP (2000). Molecular basis of muscular dystrophies. Muscle Nerve.

[40] Sarkozy A, Hicks D, Hudson J, Laval SH, Barresi R, Hilton-Jones D (2013). ANO5 gene analysis in a large cohort of patients with anoctaminopathy: confirmation of male prevalence and high occurrence of the common exon 5 gene mutation. Hum Mutat.

[41] Tang H, Mirshahidi S, Senthil M, Kazanjian K, Chen CS, Zhang KL (2014). Down-regulation of LXR/RXR activation and negative acute phase response pathways in colon adenocarcinoma revealed by proteomics and bioinformatics analysis. Cancer Biomark.

[42] Cai SY, He HW, Nguyen T, Mennone A, Boyer JL (2010). Retinoic acid represses CYP7A1 expression in human hepatocytes and HepG2 cells by FXR/RXR-dependent and independent mechanisms. J Lipid Res.

[43] Jarry J, Rioux MF, Bolduc V, Robitaille Y, Khoury V, Thiffault I (2007). A novel autosomal recessive limb-girdle muscular dystrophy with quadriceps atrophy maps to 11p13-p12. Brain.

[44] Mestas J, Hughes CCW (2004). Of mice and not men: differences between mouse and human immunology. J Immunol.

[45] Terpstra AHM (2001). Differences between humans and mice in efficacy of the body fat lowering effect of conjugated linoleic acid: role of metabolic rate. J Nutr.

[46] Yue F, Cheng Y, Breschi A, Vierstra J, Wu WS, Ryba T (2014). A comparative encyclopedia of DNA elements in the mouse genome. Nature.

[47] Grady RM, Teng HB, Nichol MC, Cunningham JC, Wilkinson RS, Sanes JR (1997). Skeletal and cardiac myopathies in mice lacking utrophin and dystrophin: A model for Duchenne muscular dystrophy. Cell.

[48] Deconinck AE, Rafael JA, Skinner JA, Brown SC, Potter AC, Metzinger L (1997). Utrophin-dystrophin-deficient mice as a model for Duchenne muscular dystrophy. Cell.

