# Supplementary material for: Genetic disruption of Ano5 in mice does not recapitulate human ANO5-deficient muscular dystrophy
Source: Skelet Muscle. 2015 Dec 21;5:43. doi: 10.1186/s13395-015-0069-z (PMC4685631; doi:10.1186/s13395-015-0069-z)
Supplement: Additional file 1: Table S1. — Primer list. Primers for genotyping, RT, and real-time PCR. [file 13395_2015_69_MOESM1_ESM.docx]

| **Primer** | **Gene name** | **Sequence (5′→3′)** |
| --- | --- | --- |
| mAno5 genotyping | Mouse Anoctamin 5 | WT-Fwd: TGAAAACCAGGAGGCAGACGAT  KO-Fwd: GGCAGGAGCAAGGTGAGATGAC  Common-Rev: GATCGCCACCTGTGCAGGCTATC |
| mAno5 set 1  RT-PCR | Mouse Anoctamin 5 | ANO5 EX3F: CTCATCCCTGAAGACTTACAGCT GAA GAC TTA CAG  ANO5 EX5R: CCACGTAGGACAGGACAAA |
| mAno5 set 2  RT-PCR | Mouse Anoctamin 5 | ANO5 EX5F: TCGACTTTGTCCTGTCCTAC TCG ACT TTG TCC TGT CCT AC  ANO5 EX7R: ACTTCAGCGTAAGTGACCAG |
| mAno5 set 3  RT-PCR | Mouse Anoctamin 5 | ANO5 12F: GACTGTGTTCTTTGCGCTCTT  ANO5 15R: GACTGACAGGCGGTATATGA |
| mAno5 set 4  RT-PCR | Mouse Anoctamin 5 | ANO5 EX15F:AAGATATCTGCCTGGATTACG  ANO5 EX17R: AATGATGGTTAGCTGGGTGG |
| mAno5 set 5  RT-PCR | Mouse Anoctamin 5 | ANO5 EX19F: CATGGGAATCCGAGTGGATG  ANO5 EX22R: AAAATTTAAACAGAAACACGACG |
| mGAPDH | Mouse Glyceraldehyde-3-Phosphate Dehydrogenase | Fwd: ACCTGCCAAGTATGATGA  Rev: GGAGTTGCTGTTGAAGTC |
| mAno1 | Mouse Anoctamin 1 | Fwd: CAGCGTCCACATCGTGAACATCT  Rev: GTATCCTCGTCATTCTCCAACTCCAG |
| mAno2 | Mouse Anoctamin 2 | Fwd: GACCACCAGAATGCCTTCACCAT  Rev: GAACCGCTGACTTGCCACTCTC |
| mAno3 | Mouse Anoctamin 3 | Fwd: TCCACCACTCAGGCTCCATTCAG  Rev: TGTTCAGATTCATCCAGGTAATCCTTGTC |
| mAno4 | Mouse Anoctamin 4 | Fwd: GCCGATGAATGCCTATGGACTCTT  Rev: AGACTCTGGCTCCGACCGATT |
| mAno5 | Mouse Anoctamin 5 | Fwd: CTGCTTCATCTATGGCTTGTTATC  Rev: GGTGTTCAGTCTCCAGTAATCA |
| mAno6 | Mouse Anoctamin 6 | Fwd: GTGTCATCAAGCCAGAGCAGGAG  Rev: TTCCAATCTTCTCGCCGTAATACTTCC |
| mAno7 | Mouse Anoctamin 7 | Fwd: TCTCATCCAACGCCAAGTCTTATTCC  Rev: GCCTCTCCTCAATGTCTTCATAGTCAG |
| mAno8 | Mouse Anoctamin 8 | Fwd: TCTGTTCTACATCGGCTTCTACCTCAA  Rev: CCTCCTCCTCCTCATTCTCCTCCT |
| mAno9 | Mouse Anoctamin 9 | Fwd: GTTATCGCCTTCACCTCTGAGTTCATC  Rev: GGTCGTCTATACATCCGTGCTCCT |
| mAno10 | Mouse Anoctamin 10 | Fwd: TTGCTTCGCCTCACTCTTCTACATT  Rev: GCCAACTGCCATACGCCAATACT |

**Supplementary Table 1: Primers for genotyping, RT and Real Time PCR**
