# Supplementary figures and images for: Genetic disruption of Ano5 in mice does not recapitulate human ANO5-deficient muscular dystrophy
Source: Skelet Muscle. 2015 Dec 21;5:43. doi: 10.1186/s13395-015-0069-z (PMC4685631; doi:10.1186/s13395-015-0069-z)

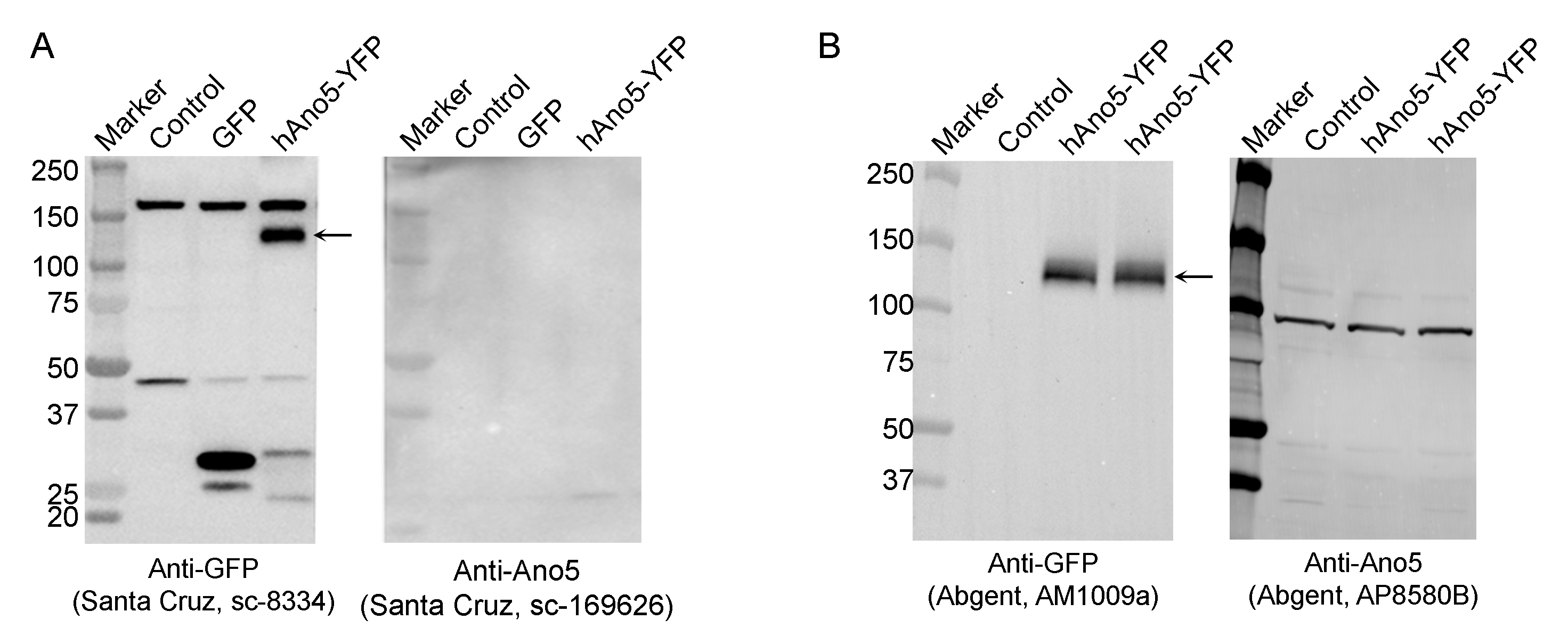

Supplement: Additional file 2: Figure S1. — Test of anti-Ano5 antibodies by Western blotting. a The anti-Ano5 antibody (sc-169626) from Santa Cruz Biotechnology did not detect any positive signal in human Ano5-expressing cell lysate, which was correctly detected by the anti-GFP antibody at the predicted size. b The anti-Ano5 antibody (AP8580B) from Abgent produced a prominent band at about 100 kDa in both negative and positive samples; however, this band is not Ano5 because the Ano5-YFP fusion protein should be around 135 kDa as correctly detected by the anti-GFP antibody. The arrows point to the correct Ano5-YFP fusion protein. These experiments were repeated at least three times. [file 13395_2015_69_MOESM2_ESM.tif]

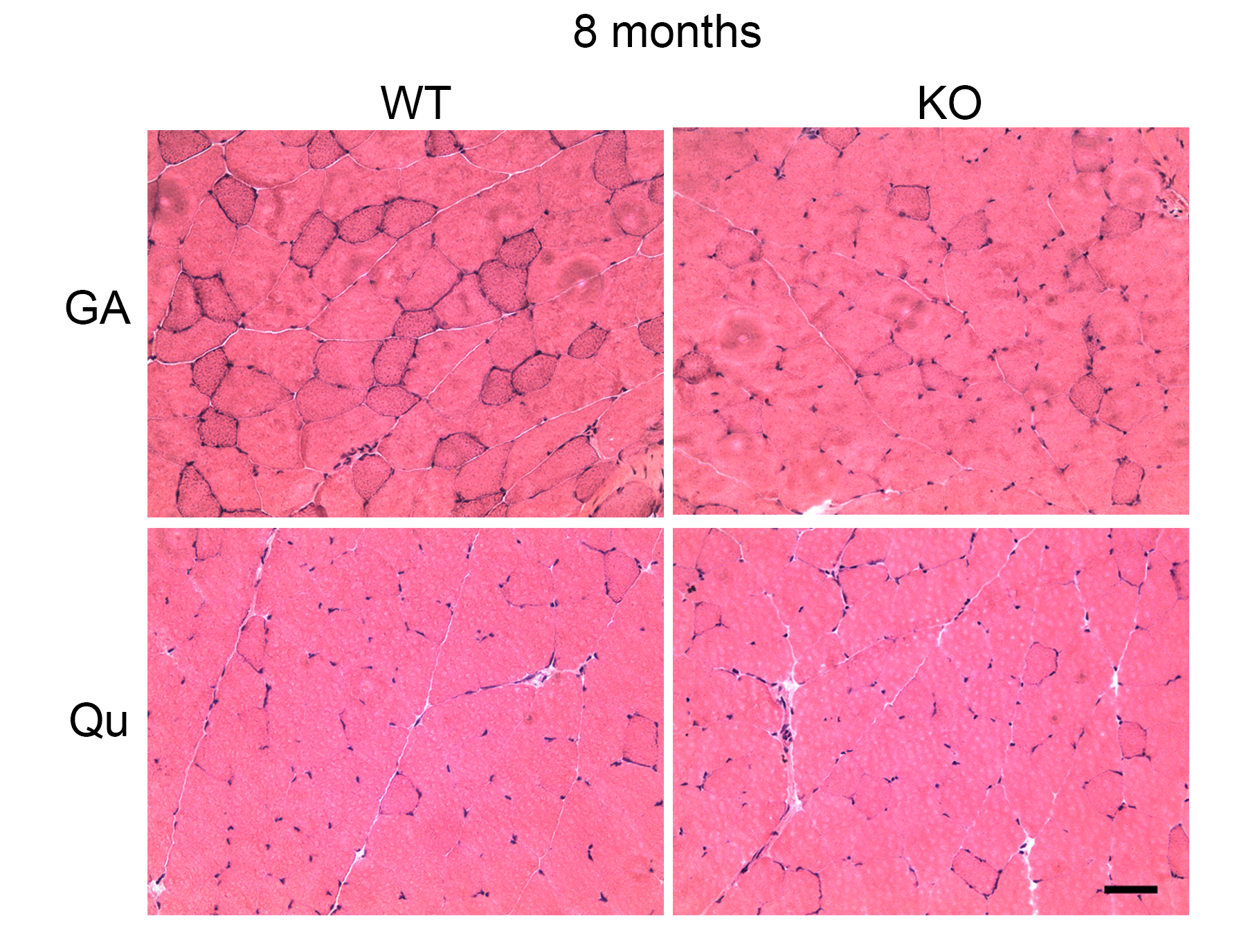

Supplement: Additional file 3: Figure S2. — H&E-stained histological sections of the gastrocnemius and quadriceps from 8-month-old mice of the indicated genotypes. Scale bar = 50 μm. The number of mice is 6–8 for each group. [file 13395_2015_69_MOESM3_ESM.tif]

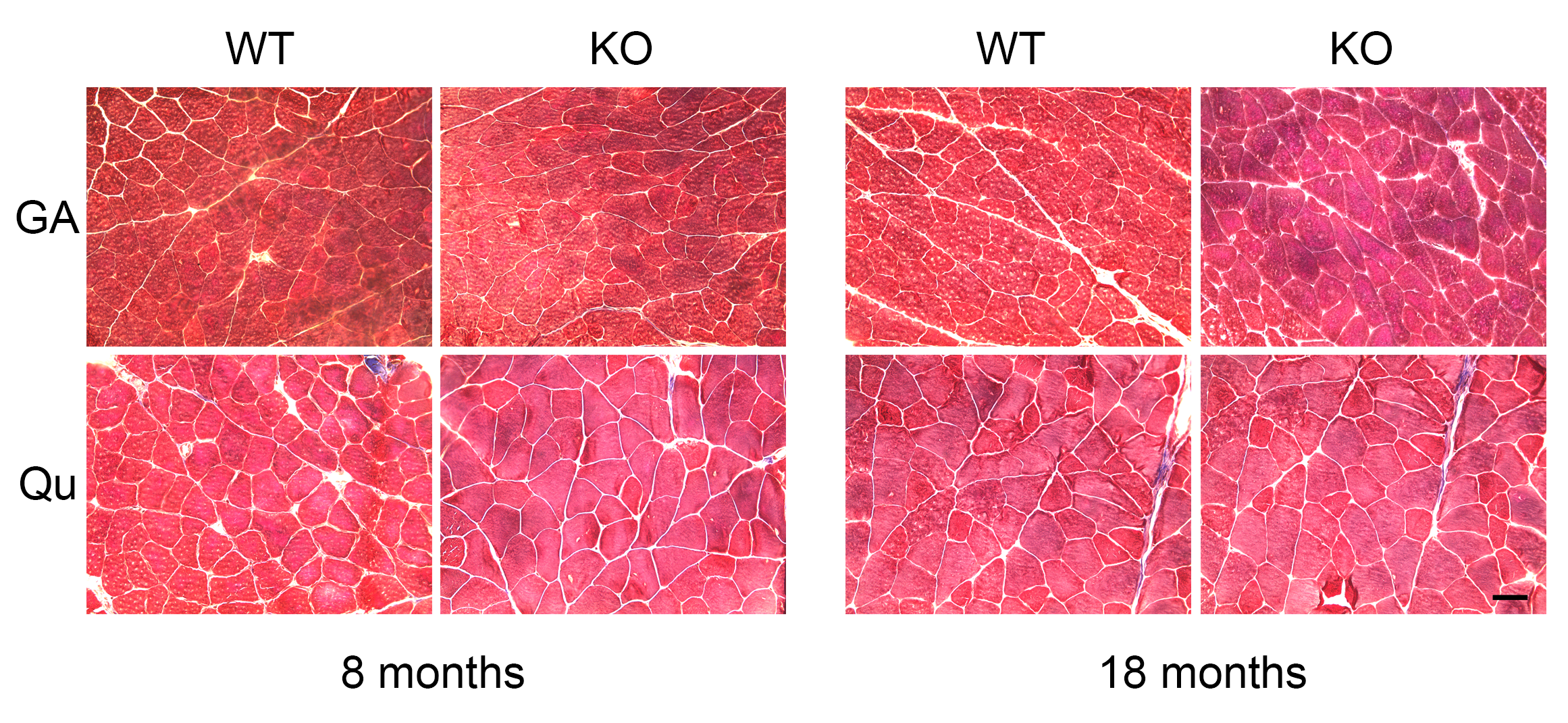

Supplement: Additional file 4: Figure S3. — Masson’s trichrome-stained histological sections of the gastrocnemius and quadriceps from 8- to 18-month-old mice of the indicated genotypes. Scale bar = 50 μm. The number of mice is 6–8 for each group. [file 13395_2015_69_MOESM4_ESM.tif]

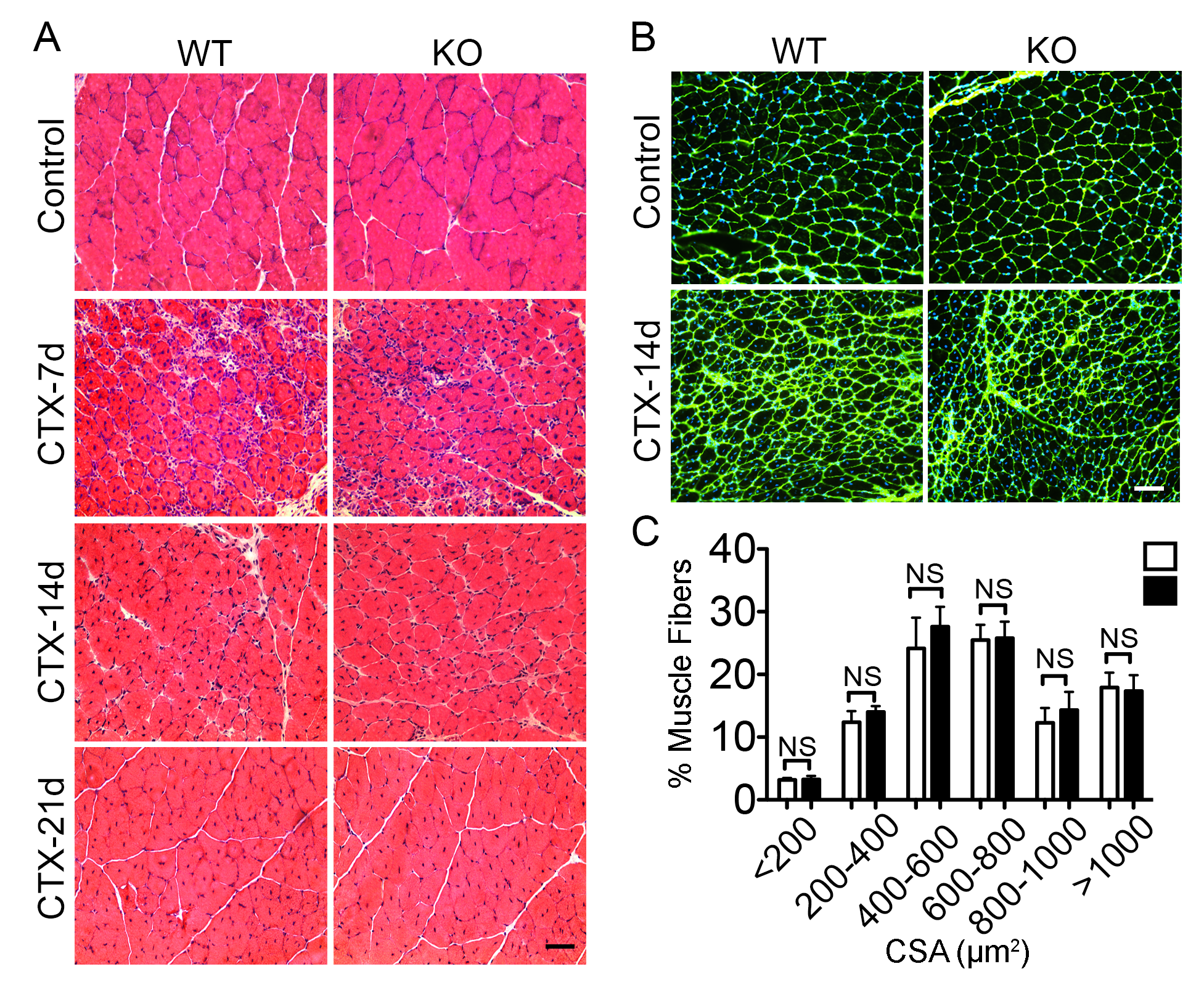

Supplement: Additional file 5: Figure S4. — Muscle regeneration after cardiotoxin injury in Ano5 KO mice. a Representative H&E-stained gastrocnemius muscle sections from WT or Ano5 KO mice after 7, 14, and 21 days post-CTX-induced injury. Scale bar = 100 μm. b Representative immunofluorescence images for caveolin-3 (green) and nucleus (DAPI, blue) staining in gastrocnemius muscle sections from WT and Ano5 KO mice at 14 days after CTX injection. Scale bar = 50 μm. c Quantitative analysis of the cross-sectional area of muscle fibers of the WT and KO mice at day 14 post-injury. ns, no statistical significance. N of mice: 3–5 for each group. [file 13395_2015_69_MOESM5_ESM.tif]

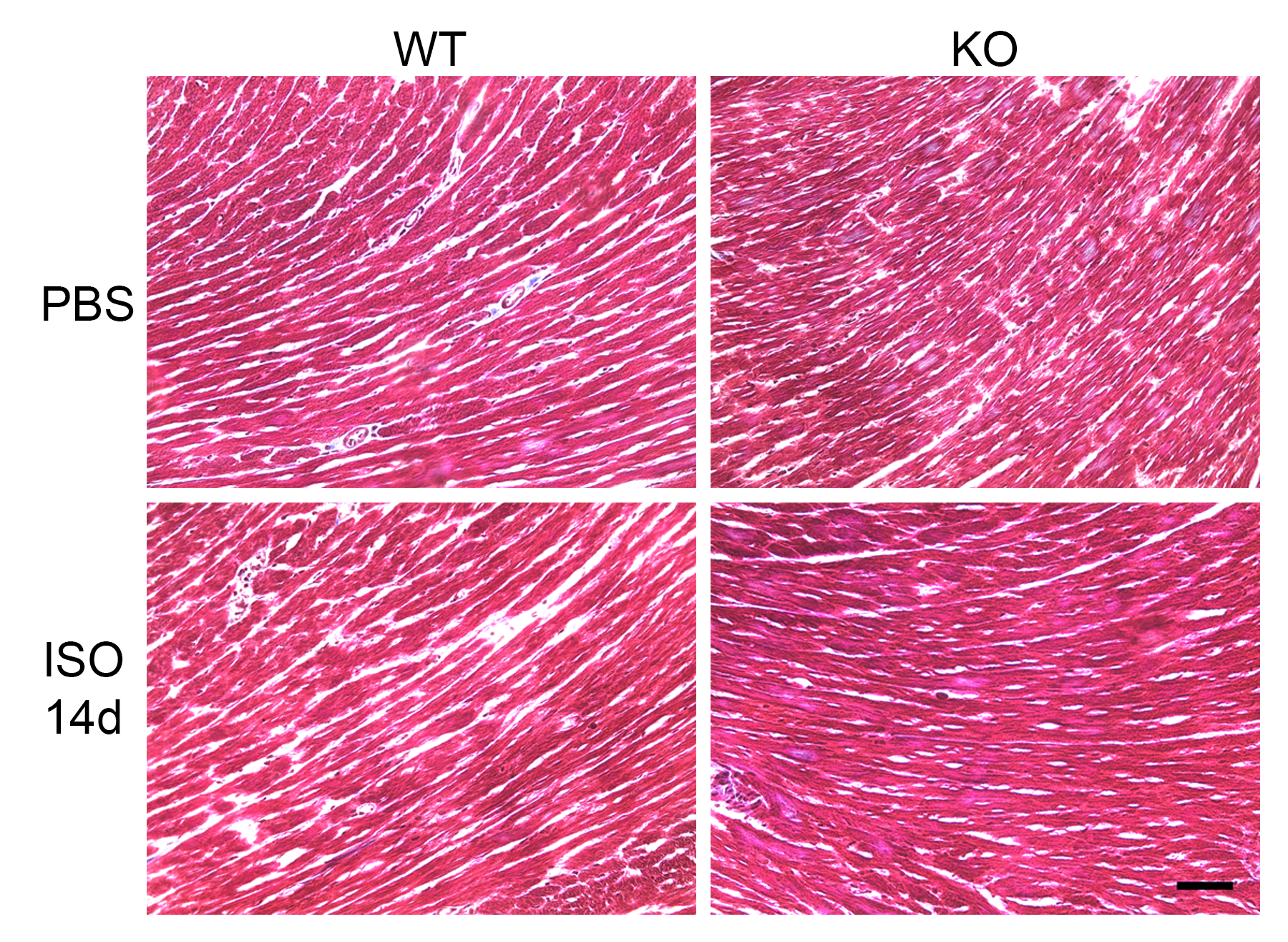

Supplement: Additional file 6: Figure S5. — Representative images of Masson’s trichrome-stained histological sections of WT and Ano5 KO hearts after PBS or ISO treatments for 2 weeks. Scale bar = 50 μm. The number of mice (18 months of age) is 6–8 for each group. [file 13395_2015_69_MOESM6_ESM.tif]
