# Supplementary material for: Genetic disruption of Ano5 in mice does not recapitulate human ANO5-deficient muscular dystrophy
Source: Skelet Muscle. 2015 Dec 21;5:43. doi: 10.1186/s13395-015-0069-z (PMC4685631; doi:10.1186/s13395-015-0069-z)
Supplement: Additional file 7: Table S2. — Echocardiographic measurements. Echocardiographic measurements in WT and Ano5 KO mice after isoproterenol injections. [file 13395_2015_69_MOESM7_ESM.docx]

**Supplementary Table 2. Echocardiographic measurements in WT and Ano5 KO mice after isoproterenol injections**

|  |  | **PBS** | | **ISO 14day** | |
| --- | --- | --- | --- | --- | --- |
| **Parameter** | **Units** | **WT** | **KO** | **WT** | **KO** |
| **Heart Rate** | BPM | 455.59±27.72 | 459.18±32.67 | 380.79±42.69* | 389.46±23.04* |
| **LVEDD** | mm | 4.18±0.64 | 4.05±0.18 | 4.47±0.62 | 4.51±0.41 |
| **LVESD** | mm | 2.48±0.39 | 2.52±0.44 | 2.82±0.51 | 2.87±0.44 |
| **Fractional Shortening** | % | 35.74±4.69 | 39.32±4.48 | 47.54±1.81* | 46.51±3.60* |
| **LV Mass** | mg | 149.13±9.02 | 134.96±4.85 | 242.46±19.19* | 234.03±15.13* |
| **LVID;d** | mm | 4.12±0.26 | 4.23±0.20 | 4.29±0.36 | 4.33±0.35 |
| **LVID;s** | mm | 2.66±0.44 | 2.63±0.36 | 2.77±0.62 | 2.71±0.48 |
| **LVPW;d** | mm | 0.86±0.14 | 0.82±0.25 | 0.99±0.15 | 0.85±0.12 |
| **LVPW;s** | mm | 1.43±0.28 | 1.45±0.15 | 1.45±0.28 | 1.46±0.10 |

BPM, beats per min; LVEDD, left ventricular end diastolic dimension; LVESD, left ventricular systolic dimension; %FS, fractional shortening; LVID;d, left-ventricular inner diameter in diastole; LVID;s, left-ventricular inner diameter in systole; LVPW;d, left-ventricular posterior wall diameter in diastole, LVPW;s, left-ventricular posterior wall diameter in systole;n=5-8/group, mean ± s.e.m. * P<0.05 comparing baseline and 2 weeks of isoproterenol treatment (paired t-test).
